# Progress on the mechanism of intestinal microbiota against colorectal cancer

**DOI:** 10.3389/fcimb.2025.1565103

**Published:** 2025-04-28

**Authors:** Guoqiang Xing, Yu Cui, Zhiyue Guo, Bing Han, Guogang Zhao

**Affiliations:** ^1^ Department of General Surgery, Tianjin Fifth Central Hospital, Tianjin, China; ^2^ College of Integrated Traditional Chinese and Western Medicine, Tianjin University of Traditional Chinese Medicine, Tianjin, China

**Keywords:** gut microbiota, colorectal cancer, mechanism of action, immune regulation, study progress

## Abstract

The intestinal microbiota plays a crucial role in the occurrence and development of colorectal cancer, and its anti - colorectal cancer mechanism has become a research hotspot. This article comprehensively expounds on the molecular mechanisms of the intestinal microbiota in anti - colorectal cancer, including aspects such as immune regulation, activation of carcinogenic signaling pathways (it should be noted that it is more reasonable to be “inhibition of carcinogenic signaling pathways”), metabolite - mediated effects, and maintenance of intestinal barrier function. At the same time, it explores the roles and potential mechanisms of intervention methods such as probiotic supplementation therapy, immunotherapy, and fecal microbiota transplantation. In addition, it analyzes the impact of the intestinal flora on the therapeutic efficacy of colorectal cancer. The existing research results are summarized, and the future research directions are prospected, with the aim of providing new theoretical bases and treatment ideas for the prevention and treatment of colorectal cancer.

## 1 Introduction

Colorectal cancer, as one of the malignant tumors with high incidence and mortality rates globally, is currently ranked third in incidence and second in mortality according to the latest global cancer burden data released by the International Agency for Research on Cancer (IARC) of the World Health Organization in 2020. It accounts for 10% and 9.4% of the total cases of cancer incidence and mortality, respectively, posing a significant threat to human health and quality of life (Smith et al., 2024; Huang ZM. et al., 2024). Despite certain advancements in diagnostic and therapeutic approaches, there remains an urgent need to further investigate its pathogenesis and effective prevention and treatment strategies (Marcellinaro et al., 2023). In recent years, the role of the gut microbiota in human health and disease has gradually emerged as a focal point of medical research, with particular attention being paid to its relationship with colorectal cancer (Janney et al., 2020). The human gastrointestinal tract harbors trillions of microorganisms, which form a complex and delicate ecosystem with the host, playing a crucial role in maintaining normal physiological functions of the gut, participating in nutrient metabolism, and regulating the immune system (Tremaroli and Bäckhed, 2024). A substantial body of clinical and basic research evidence indicates that alterations in the composition and function of the gut microbiota are closely linked to the occurrence and progression of colorectal cancer (Garrett, 2019; Yan et al., 2017). Compared to healthy individuals, colorectal cancer patients exhibit a marked dysbiosis in their gut microbiota, characterized by a significant increase in the abundance of certain pathogenic microorganisms and a relative decrease in beneficial microbes (Wang and Dong, 2021). This alteration in microbial communities may influence the disease progression of colorectal cancer through various mechanisms, including but not limited to the induction of chronic inflammatory responses, the production of carcinogenic metabolites, the impact on intestinal barrier integrity, and the modulation of the host immune response (Chassaing et al., 2014). Therefore, a comprehensive understanding of the mechanisms by which gut microbiota exerts anti-colorectal cancer effects is of paramount theoretical and clinical significance for elucidating the pathogenesis of colorectal cancer, developing novel diagnostic biomarkers, and innovating therapeutic strategies.

**Figure 1:**
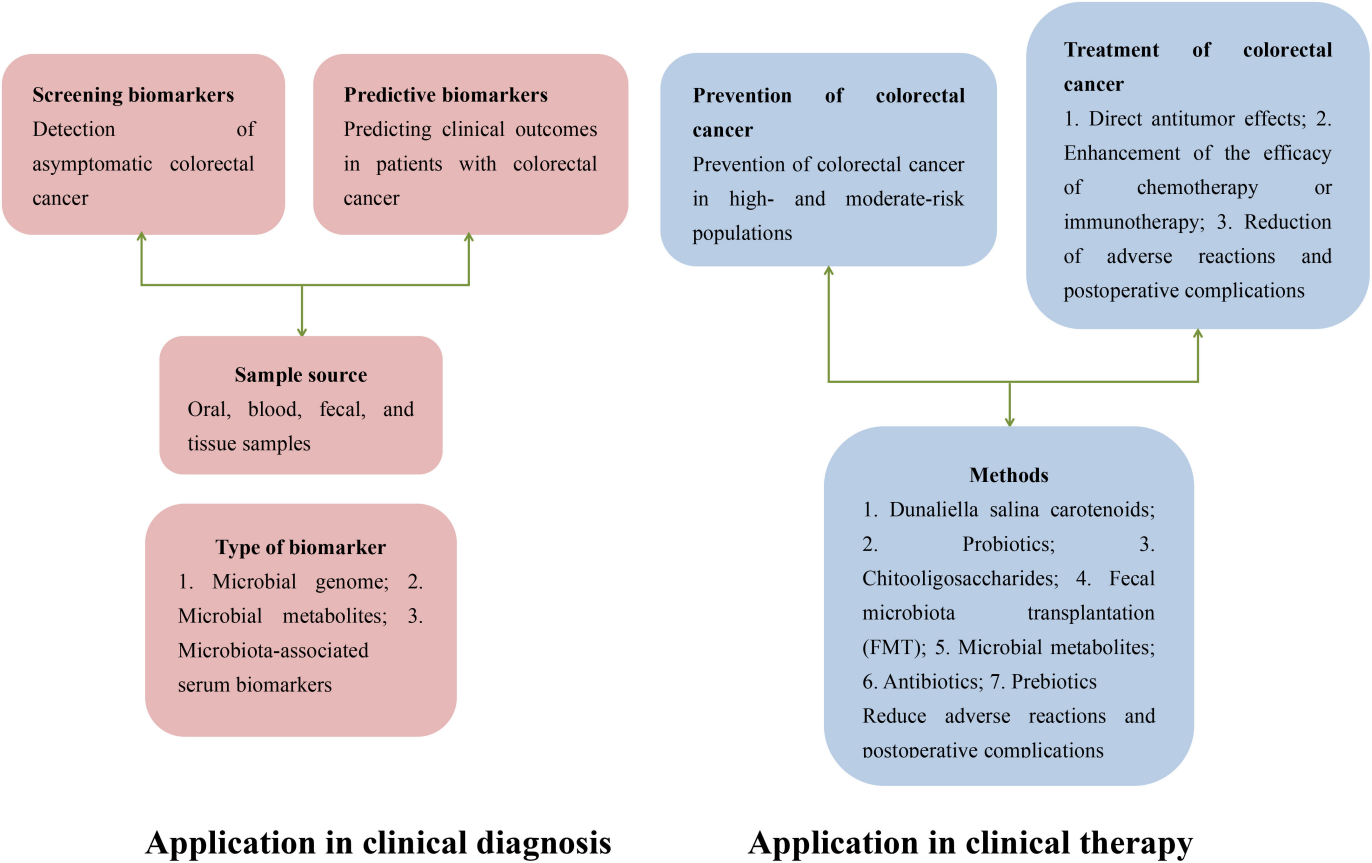
Potential applications of gut microbiota in the diagnosis and treatment of colorectal cancer.

**Figure 1** illustrates the potential applications of gut microbiota in the diagnosis and treatment of colorectal cancer (CRC). The gut microbiota holds significant value in the early diagnosis of CRC. Specific microbial biomarkers, such as *Fusobacterium nucleatum* and *Bacteroides fragilis*, are markedly enriched in CRC patients and can be detected in fecal samples, offering a non-invasive diagnostic approach. Additionally, compositional shifts in the microbial community—including reduced microbial diversity and dysbiosis of specific bacterial taxa—may serve as novel biomarkers for early CRC screening and risk stratification. In therapeutic contexts, the gut microbiota demonstrates broad prospects for CRC management. On one hand, microbiota-mediated modulation of the host immune system can influence the tumor microenvironment, thereby enhancing the efficacy of immunotherapy. For instance, specific probiotics (e.g., *Bifidobacterium* and *Lactobacillus*) have been shown to activate antitumor immune responses and potentiate the therapeutic effects of immune checkpoint inhibitors, such as PD-1/PD-L1 inhibitors. On the other hand, microbial-derived metabolites, including short-chain fatty acids (SCFAs), exert direct antitumor effects by suppressing proliferation or inducing apoptosis in neoplastic cells. Furthermore, microbiota-targeted personalized therapies—such as fecal microbiota transplantation (FMT)—are under active investigation, aiming to optimize treatment outcomes and mitigate adverse effects through strategic modulation of gut microbial composition. This article aims to provide a comprehensive review of the research progress regarding the mechanisms by which gut microbiota contributes to anti-colorectal cancer effects, with the intention of offering valuable references and insights for further exploration and clinical application in this field.

## 2 Overview of the gut microbiota

The gut microbiota is a vast and intricate ecosystem residing within the human gastrointestinal tract, primarily composed of bacteria, fungi, viruses, and archaea, with bacteria dominating in both quantity and function. In terms of sheer numbers, the gut microbiota is extraordinarily abundant, estimated to contain approximately 10^14 microbial individuals, with a total gene count exceeding 100 times that of the human genome, which underscores its potential influence on human physiological processes (Kim and Lee, 2022). The diversity of gut bacteria is significant, with over 1,000 identified species belonging to various phyla, including Firmicutes, Bacteroidetes, Proteobacteria, and Actinobacteria, among which Firmicutes and Bacteroidetes account for the highest proportions, approximately 90%. These bacteria are unevenly distributed throughout the gastrointestinal tract, with their numbers and types gradually increasing from the stomach to the colon, where the colon represents the most densely populated area, containing up to 10^11 to 10^12 bacterial cells per gram of intestinal content (Gao et al., 2017). The gut microbiota and the host have co-evolved over a long period, establishing a close and complex symbiotic relationship that participates in numerous physiological functions. During digestion, they assist in breaking down complex carbohydrates, proteins, and fats found in food; related studies have indicated that colonic probiotics can ferment dietary fibers to produce short-chain fatty acids, thereby providing energy to the host. Additionally, they play a crucial role in vitamin synthesis (such as vitamin K and B vitamins) and mineral absorption, ensuring the host’s nutritional needs are met (Fusco et al., 2024). Furthermore, research has confirmed that the gut microbiota actively participates in the development and maturation of the human immune system, regulating the balance of immune responses through interactions with intestinal epithelial cells and immune cells, which enables the body to fend off pathogenic invasions while preventing excessive inflammatory responses that could harm the organism, thereby maintaining a stable intestinal environment and laying the foundation for human health (Fusco et al., 2024). However, this balance, once disrupted, may be associated with the occurrence and progression of various diseases, including colorectal cancer.

## 3 The association between gut microbiota and colorectal cancer

A substantial body of research indicates that changes in the composition and function of the gut microbiota are closely associated with the occurrence and progression of colorectal cancer (Wong and Yu, 2023). In the gut microbiota of colorectal cancer patients, a pronounced dysbiosis has been observed. The abundance of certain pathogenic bacteria has significantly increased; studies have confirmed that the levels of specific virulent strains of *Fusobacterium nucleatum*, *Escherichia coli*, and *Bacteroides fragilis* have risen markedly (Quaglio et al., 2022). *Fusobacterium nucleatum* can bind to receptors on the surface of colorectal cancer cells through its surface adhesins, promoting the proliferation, migration, and invasion of cancer cells, and it accumulates in tumor tissues, correlating with poor prognosis (Góralczyk-Bińkowska et al., 2022). Certain pathogenic strains of *Escherichia coli* can produce genotoxins, such as colibactin, which can induce DNA damage in host cells, leading to genomic instability and increasing the risk of colorectal cancer (Ling et al., 2022). Concurrently, the abundance of beneficial bacteria tends to decrease in the intestines of colorectal cancer patients (Wang et al., 2023b). Research has shown that beneficial bacteria, such as *Bifidobacterium* and *Lactobacillus*, play crucial roles in maintaining intestinal barrier integrity, regulating immunity, and inhibiting the growth of harmful bacteria. A reduction in their numbers weakens intestinal protective functions, creating favorable conditions for tumorigenesis (Sen et al., 2021). The gut microbiota also influences the progression of colorectal cancer through its metabolic products (Fan et al., 2023). Relevant studies have indicated that secondary bile acids produced by the gut bacterium *Fusobacterium nucleatum* exhibit carcinogenic effects at high concentrations, inducing intestinal inflammation and cell proliferation (Schneider et al., 2024). In contrast, short-chain fatty acids produced by the fermentation of beneficial bacteria, such as butyrate, exhibit anti-cancer effects by inhibiting tumor cell growth, inducing apoptosis, and modulating immunity. The imbalance of metabolic products from the gut microbiota plays a critical role in the development of colorectal cancer (Feizi et al., 2023).

**Table 1** elaborates the multifaceted roles and functional mechanisms of gut microbiota in colorectal carcinogenesis. *Fusobacterium nucleatum*: Aggregates in CRC tissues, activating NF-κB and Wnt signaling pathways upon colonization. NF-κB, a pivotal transcription factor, promotes cell proliferation, anti-apoptosis, and inflammation-associated gene expression upon activation, fostering a permissive microenvironment for cancer cell survival. Aberrant Wnt signaling disrupts normal cellular differentiation and proliferation, driving uncontrolled neoplastic growth. *Escherichia coli*: Functions as a driver through inflammation mediation, inducing chronic mucosal damage and impaired repair that establishes a pro-carcinogenic inflammatory milieu. Reactive oxygen species generated during inflammation exacerbate DNA damage and mutagenesis, accelerating malignant transformation. *Bacteroides fragilis*: Produces enterotoxins that degrade E-cadherin, compromising intercellular junctions and facilitating metastasis. Concurrently, it induces Th17/IL-17-mediated inflammation to promote tumor progression. *Bifidobacterium*: Exerts protective effects by reducing β-glucuronidase activity, thereby inhibiting the conversion of procarcinogens into active carcinogens. *Lactobacillus*: Mitigates intestinal acidification through lactate reduction while activating Toll-like receptors to enhance anti-tumor immunity. *Helicobacter pylori*: Elevated in CRC patients, its vacuolating cytotoxin damages epithelial integrity and potentiates carcinogen susceptibility. *Enterococcus faecalis*: Generates extracellular superoxide that induces DNA damage and dysregulated proliferation. Eubacterium rectale and *Clostridium septicum*: Their depletion reduces protective butyrate/short-chain fatty acid production, impairing anti-inflammatory responses and epithelial protection, thereby elevating CRC risk.

**Table 1:** The impact and functions of gut microbiota on the occurrence of colorectal cancer.

Microorganisms	Impact on Colorectal Cancer	Functions
*Fusobacterium nucleatum*	Increased Abundance in Colorectal Cancer	Accumulation in Colorectal Cancer, Activating NF-κB and Wnt Signaling Pathways
*Escherichia coli*	Oncogenic Role	Mediating Inflammation
*Bacteroides fragilis*	Oncogenic Role	Toxic Products, Promoting E-Cadherin Degradation, Inducing Th17/IL-17 Inflammatory Response
*Bifidobacterium*	Protective Role	Reducing β-Glucuronidase Activity
*Lactobacillus*	Protective Role	Decreasing Lactate Production, Activating Toll-like Receptors
*Helicobacter pylori*	Increased Abundance in Colorectal Cancer	Producing Various Functional Vacuolating Toxins
*Enterococcus faecalis*	Oncogenic Role	Generating Extracellular Peroxides Leading to DNA Damage
*Faecalibacterium prausnitzii*	Decreased Abundance in Colorectal Cancer	Producing Butyrate
*Clostridium septicum*	Decreased Abundance in Colorectal Cancer	Short-chain fatty acids (SCFAs)

Moreover, the abnormal interaction between the gut microbiota and the host immune system is also associated with colorectal cancer. Dysbiosis may lead to alterations in the intestinal immune microenvironment, resulting in chronic inflammation; the prolonged stimulation of colonic epithelial cells by inflammatory factors can promote cellular carcinogenesis, while the immune system’s ability to surveil and eliminate tumor cells may also be compromised, further facilitating the progression of colorectal cancer. These findings underscore the significant role of the gut microbiota in the pathogenesis of colorectal cancer and provide critical insights for further exploration of its mechanisms and the development of therapeutic strategies.

## 4 Molecular mechanisms of the gut microbiota in the anticancer activity against colorectal cancer

### 4.1 Host genetic variations

In the association between the gut microbiota and colorectal cancer, host genetic variations play a pivotal role. The microbiota can influence host gene expression through various mechanisms, thereby affecting the onset and progression of colorectal cancer. Relevant studies have indicated that bacteria from the genus Streptococcus are significantly enriched in colorectal cancer patients with KRAS gene mutations (Pant et al., 2023). Additionally, other researchers have confirmed that the abundance of non-toxigenic *Bacteroides fragilis* is associated with a CpG island methylation phenotype (CIMP)-high and microsatellite instability (MSI)-high status, while the abundance of enterotoxigenic *Bacteroides fragilis* correlates with CIMP-high and BRAF mutations. Furthermore, studies have shown that short-chain fatty acids can enter host cells and act as signaling molecules for intracellular transcription factors, inhibiting the expression of genes associated with pro-carcinogenic signaling pathways such as NF-κB, while reducing the activation of genes related to inflammatory responses and tumor cell proliferation (Zhao and Zou, 2023). Simultaneously, the microbiota may also influence the host’s DNA methylation patterns and histone modification states. Research has demonstrated that beneficial bacteria can regulate the activity of DNA methyltransferases, leading to the demethylation of specific tumor suppressor genes and restoring their normal expression, thereby exerting tumor-suppressive functions (Yu et al., 2024). In terms of histone modifications, microbial metabolites can alter the acetylation and methylation levels of histones, reshaping chromatin structure and resulting in changes in the expression of genes related to cell cycle regulation and apoptosis, thereby enhancing the host cells’ resistance to tumorigenesis. These changes at the level of host genetics reveal the complex and intricate molecular regulatory network of the gut microbiota in the anticancer process against colorectal cancer, providing an important theoretical foundation for further exploration of prevention and treatment strategies.

### 4.2 Activation of carcinogenic signaling pathways

Clinical studies have suggested that the transformation from normal cells to tumor cells is caused by the disruption of regulatory mechanisms within cellular pathways (Ronen et al., 2024). As shown in **Table 2**, international researchers have confirmed a relationship between host signaling pathways and intratumoral bacteria, revealing that colorectal cancer is associated with the modulation of the peripheral blood PI3K-Akt signaling pathway (Chen Y. et al., 2024). In a study involving 96 patients with advanced colorectal cancer, it was indicated that downregulation of the PI3K-Akt signaling pathway could improve the clinical status of colorectal cancer. Numerous pathogens transduce the PI3K-Akt signaling pathway by directly interacting with surface receptors on colonic epithelial cells. Furthermore, other scholars have demonstrated a close association between the Notch1 signaling pathway and colorectal cancer (Png et al., 2024). In an experiment utilizing azoxymethane/dextran sulfate sodium-induced inflammation-associated colorectal cancer mice, which were divided into a control group, a model group, and a ginsenoside Rg-3 group, each consisting of 16 mice, it was concluded that low expression of the Notch1 signaling pathway is related to enhanced angiogenesis, thereby inhibiting the progression of azoxymethane/dextran sulfate sodium-induced colorectal cancer. Research has also shown that Fusobacterium, through its surface adhesin FadA, binds to E-cadherin on colonic epithelial cells, activating the Wnt/β-Catenin signaling pathway and mediating pro-tumor effects (Zhao et al., 2024). **Table 2** elucidates the intricate interactions between gut microbiota and carcinogenic signaling pathways. The intestinal microbiota influences oncogenic pathways through multifaceted mechanisms, encompassing microbial metabolites, inflammatory responses, and immunomodulatory effects. Specifically, certain microbial metabolites such as short-chain fatty acids (SCFAs) and secondary bile acids demonstrate regulatory capacities—either directly or indirectly—over pivotal carcinogenic signaling pathways including Wnt/β-catenin, PI3K/AKT, and NF-κB. Furthermore, gut dysbiosis may induce chronic inflammatory states that subsequently activate STAT3 and MAPK signaling cascades, thereby fostering tumorigenesis. Conversely, probiotic species such as *Lactobacillus* and *Bifidobacterium* exhibit antitumor properties through suppression of these pathological signaling pathways.

**Table 2:** The relationship between gut microbiota and carcinogenic signaling pathways.

Microbial Strains	Signaling Pathways	Biological Effects	Literature
*Polybacterium*	Notch signaling pathway	Mediating the Self-Renewal of Colorectal Cancer Stem Cells (CCSCs)	(Wong and Yu, 2023)
	MAPK(JNK)-AP1 signaling pathway	Upregulation of MMP-7 Expression Induces Metastasis in Colorectal Cancer Cells	(Quaglio et al., 2022)
	Alpk1-NF-κB signaling pathway	Upregulation of the Adhesion Molecule ICAM1 Expression Promotes Metastasis	(Wang et al., 2023b)
	Wnt signaling pathway	Inducing Metastasis in Colorectal Cancer Cells	(Feizi et al., 2023)
*Anaerobic Digestion Streptococcus*	PI3K-Akt signaling pathway	Myeloid-derived suppressor cells, tumor-associated macrophages, and tumor-associated neutrophils are significantly increased, driving the progression of colorectal cancer.	(Chen Y. et al., 2024)
*Porphyromonas gingivalis*	MAPK/ERK signaling pathway	Promoting the Proliferation of Colorectal Cancer Cells	(Liu et al., 2021a)

### 4.3 Immunoregulatory effects

**Figure 2:**
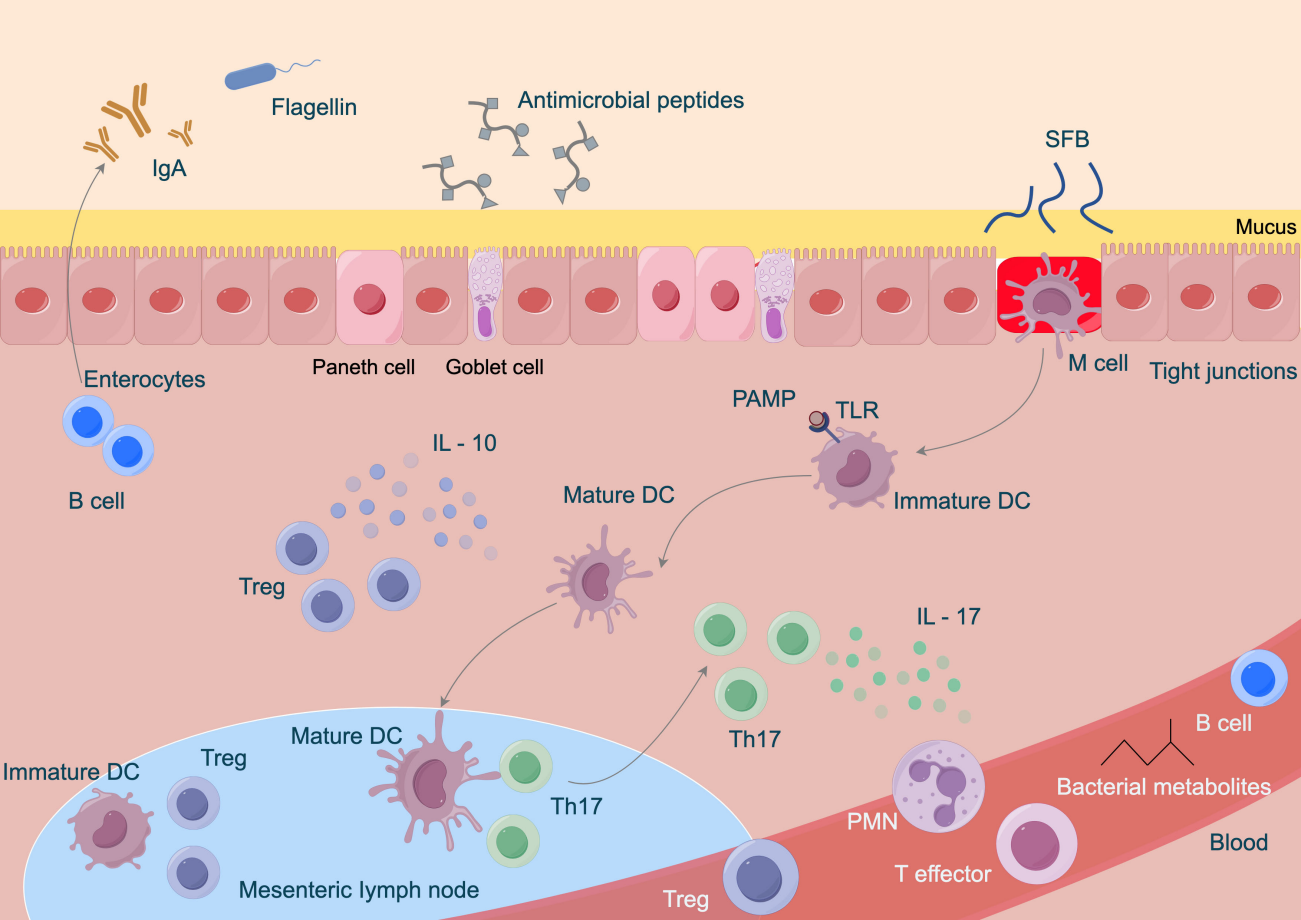
Immune modulation relationships of gut microbiota in colorectal cancer.

The gut microbiota exerts anti-colorectal cancer effects through various immune modulation pathways. On one hand, certain beneficial bacteria can stimulate the maturation and differentiation of immune cells within the intestine (Wegierska et al., 2022). Research has confirmed that *Bifidobacterium* and others can activate dendritic cells, enhancing their antigen-presenting capabilities, thereby inducing T lymphocyte differentiation into effector CD8+ T cells with anti-tumor activity to kill colorectal cancer cells (Mhanna et al., 2024). Furthermore, the microbiota can regulate the secretion of immune-related cytokines. For instance, some *Lactobacillus* strains can promote the production of anti-inflammatory factors such as IL-10 while inhibiting the excessive expression of pro-inflammatory factors like TNF-α and IL-6, creating an immune microenvironment conducive to anti-cancer activity and reducing the risk of inflammation-induced cancer (Liu et al., 2021a). The immune modulation relationships are illustrated in **Figure 2**. Related studies indicate that collecting fecal samples from infants aged 30 to 35 days and extracting *Bifidobacterium* bifidum, *Bifidobacterium* longum, and *Bifidobacterium* infantis, followed by co-culturing with dendritic cells obtained from the infants’ cord blood, revealed that, except for *Bifidobacterium* infantis, the other *Bifidobacterium* strains could enhance effector CD8+ T cells and increase IL-10 levels (Chen and Wang, 2022; Zhu et al., 2022; D’Amico et al., 2023; Lee and Chiu, 2024). Additionally, the gut microbiota is involved in maintaining the integrity of the intestinal mucosal barrier. Studies have found that during Gram-positive bacterial infections, the gut microbiota can induce the production of Small Proline-Rich Protein 2A (SPRR2A), which disrupts the cell membrane of Gram-positive bacteria, preventing such bacteria from breaching the intestinal barrier (Settanni et al., 2021; Li and Roy, 2023; Liu et al., 2023; Masenga and Kirabo, 2023; Suslov et al., 2024). Simultaneously, the gut microbiota can induce intestinal epithelial cells to secrete mucus and tight junction proteins, blocking the invasion of harmful substances and pathogens, thereby preventing abnormal immune activation and indirectly hindering the occurrence and development of colorectal cancer, contributing to the fight against colorectal cancer at the level of immune modulation.

### 4.4 Metabolite-mediated effects

**Figure 3:**
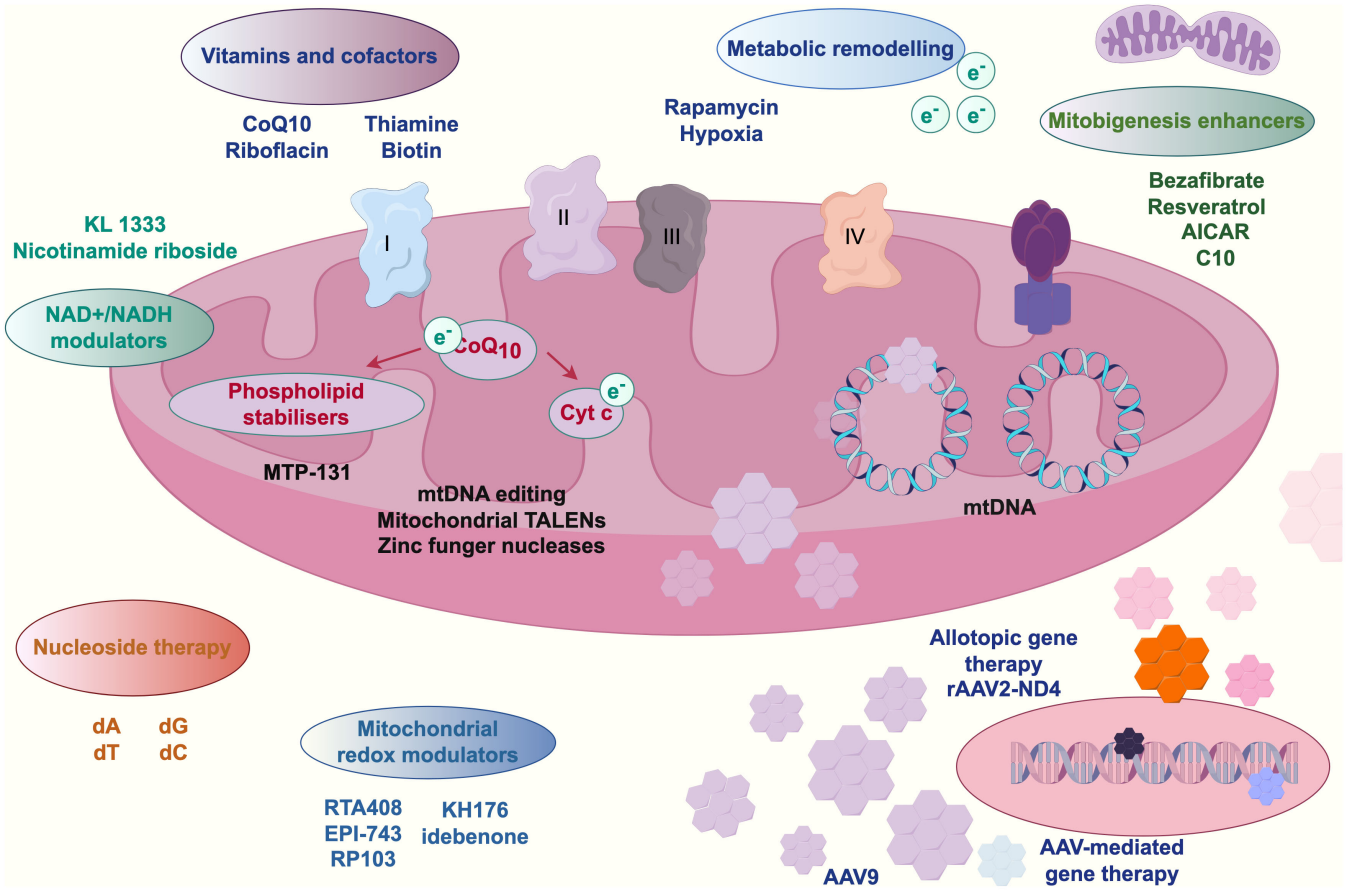
Mediating relationships of metabolites of gut microbiota in colorectal cancer.

Empirical studies (Zhou et al., 2021; Bastings et al., 2023; Pérez-Morales et al., 2024) have confirmed that during colorectal carcinogenesis, gut microbiota-derived metabolites play pivotal mediating roles. Mitochondria, as central hubs of cellular energy metabolism, exhibit functional interdependence with these metabolites. Substances such as nicotinamide adenine dinucleotide (NAD+)-nicotinamide participate in mitochondrial physiological signaling and energy metabolism. Intestinal microbiota dysbiosis elevates harmful stimuli, potentially triggering mitochondrial dynamics alterations—including aberrant fission-fusion equilibrium—that perturb cellular metabolism. Kynurenine, a microbial metabolite, engages in metabolic reprogramming by interacting with mitochondrial transport proteins (e.g., mitochondrial carrier homolog 1) to modulate intracellular trafficking and bioenergetics, thereby promoting colorectal cancer cell proliferation and survival. Concurrently, microRNAs (miRNAs) regulate mitochondrial functionality and cellular metabolism through gene expression modulation (Badgeley et al., 2021). Gut microbiota-derived vitamins and cofactors—including coenzyme Q10, riboflavin, and biotin—are indispensable for mitochondrial function maintenance, participating in oxidative phosphorylation pathways; their metabolic dysregulation may induce mitochondrial dysfunction and drive oncogenesis (Wang et al., 2023a; Chandrasekaran et al., 2024; Lamaudière et al., 2024). Emerging interventions such as adeno-associated virus (AAV)-mediated gene therapy and mitochondria-targeting nanoparticles offer novel strategies to counteract microbiota-metabolite-mediated colorectal cancer progression. **Figure 3** schematically illustrates this metabolite-mediated mechanism of gut microbiota in colorectal carcinogenesis.

### 4.5 Induction of inflammatory responses

**Figure 4:**
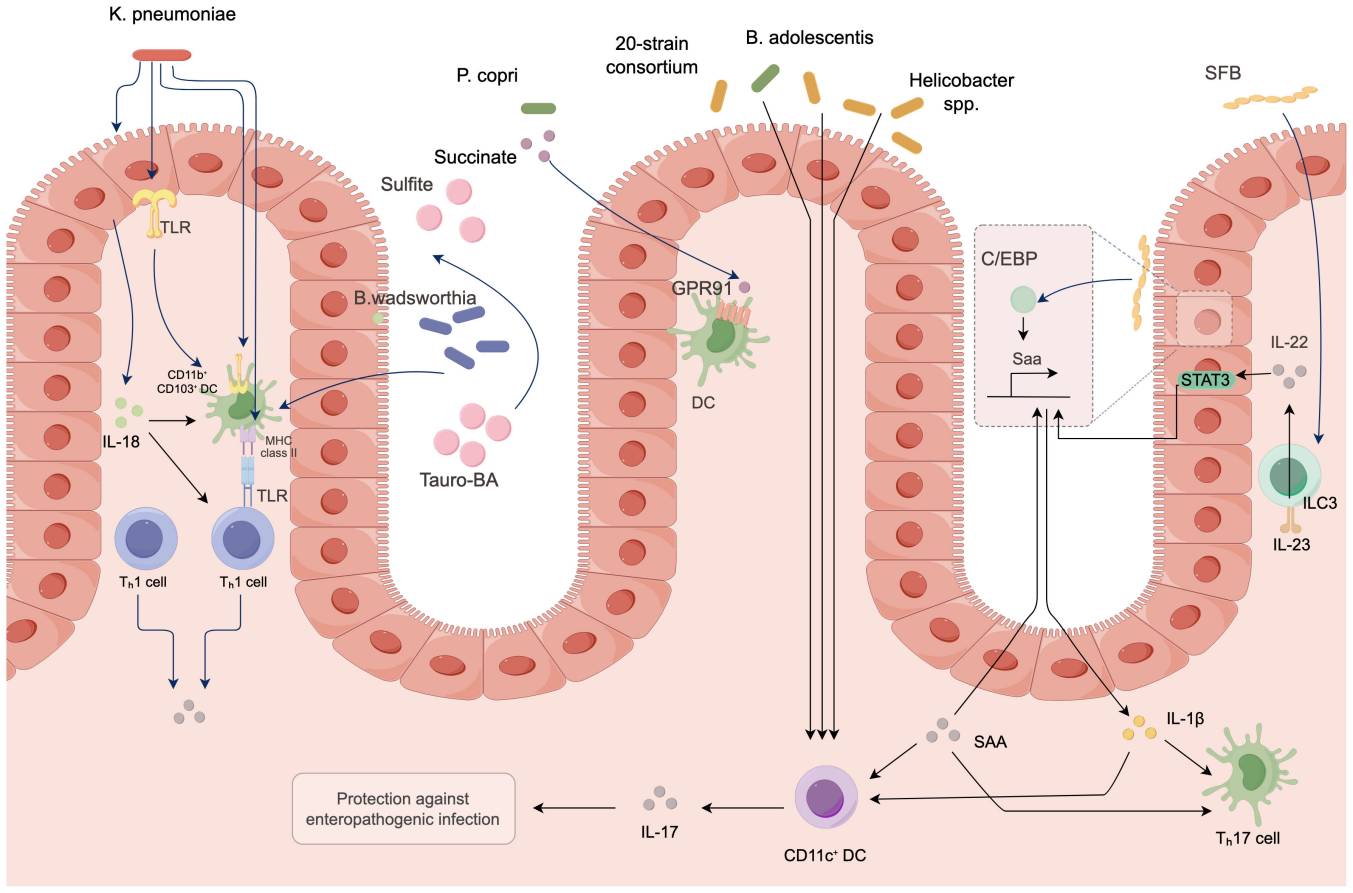
Molecular mechanisms of gut microbiota in colorectal cancer.

Under normal circumstances, the gut microbiota maintains immune homeostasis; however, under certain conditions, it can also induce inflammatory responses that promote colorectal cancer (Li et al., 2021). Some pathogenic bacteria produce endotoxins such as lipopolysaccharides, which activate pattern recognition receptors on the surface of host immune cells. Research has confirmed that Toll-like receptors can initiate inflammatory signaling pathways such as NF-κB (Kaul et al., 2024). When the NF-κB pathway is activated, it releases a substantial amount of inflammatory cytokines, including interleukin-6 (IL-6), tumor necrosis factor-alpha (TNF-α), and interleukin-17 (IL-17). A sustained inflammatory environment can lead to DNA damage, abnormal cell proliferation, and the recruitment of immune cells, creating a microenvironment conducive to tumor cell proliferation and metastasis, thereby promoting the occurrence and progression of colorectal cancer. The mechanisms of action are illustrated in **Figure 4**. Furthermore, foundational experimental research has demonstrated that a study involving 50 SD rats, which constructed a colorectal cancer model, randomly assigned them into a model group, a probiotic group, a gut microbiota transplantation group, and a gut microbiota transplantation plus probiotic group, with 10 rats in each group. The results indicated that gut microbiota transplantation combined with probiotics significantly alleviated the inflammatory response in colorectal cancer rats. This leads to the conclusion that there is an association between inflammatory responses and gut microbiota, which can drive the progression of colorectal cancer (Xu et al., 2024).

### 4.6 Maintaining gut barrier function

The integrity of intestinal barrier function is crucial for the prevention of colorectal cancer, and the gut microbiota plays a key role in maintaining this barrier function (Melamed et al., 2022; Gervasi and Mandalari, 2023; Luo et al., 2023). The gut microbiota enhances tight junctions between intestinal epithelial cells through various mechanisms. Studies have shown that beneficial bacteria such as *Bifidobacterium* and *Lactobacillus* acidophilus can stimulate intestinal epithelial cells to secrete tight junction proteins, leading to increased expression of associated proteins such as ZO-1 and occludin, which help to reinforce the connections between epithelial cells and form a tight physical barrier that prevents harmful substances, bacteria, and toxins from crossing the intestinal mucosa into the bloodstream, thereby reducing the risk of intestinal inflammation and tumorigenesis. Additionally, the gut microbiota is involved in the formation and maintenance of the mucus layer. Research has indicated that the mucus secreted by intestinal goblet cells is an important component of the intestinal barrier, and the gut microbiota can regulate the secretion state of mucus, making it more viscous to effectively resist pathogen invasion, reduce damage to intestinal epithelial cells, and mitigate inflammatory responses, thereby providing a stable internal environment for intestinal epithelial cells and inhibiting the occurrence and progression of colorectal cancer (Xu et al., 2020; Cortés et al., 2021; Matsuzaki et al., 2023).

## 5 Molecular mechanisms by which gut microbiota influences the efficacy of colorectal cancer treatment

### 5.1 Probiotic supplementation therapy

Probiotic supplementation therapy, as one of the strategies for modulating the gut microbiota to combat colorectal cancer, has garnered significant attention in recent years, with its molecular mechanisms of action encompassing multiple dimensions.

#### 5.1.1 In terms of immune modulation

Many probiotics, such as *Bifidobacterium* and *Lactobacillus* acidophilus, can stimulate the development and maturation of gut-associated lymphoid tissue, thereby enhancing the activity of immune cells. Research has confirmed that a study involving 110 gastric cancer patients divided them into a control group and a probiotic group. The results indicated that the probiotic group exhibited a significant enhancement in their immune function. Consequently, it was concluded that the supplementation of probiotics in gastric cancer patients with gastrointestinal dysfunction could promote the maturation and differentiation of dendritic cells (DCs), upregulate the expression of surface co-stimulatory molecules, and more effectively present antigens to T lymphocytes, activating CD4+ and CD8+ T cells, thereby enhancing the body’s anti-tumor immune response and inducing apoptosis in cancer cells or inhibiting their proliferation (Yang et al., 2022). Additionally, probiotics can modulate the cytokine secretion profile, increasing the production of anti-inflammatory cytokines such as IL-10 while suppressing the release of pro-inflammatory cytokines like IL-6 and TNF-α, thereby maintaining the stability of the intestinal immune microenvironment and reducing the risk of inflammation-related colorectal cancer (Marra et al., 2021; Koga, 2022; Zhu and Ding, 2024).

#### 5.1.2 In terms of maintaining intestinal barrier function

Probiotics can induce intestinal epithelial cells to secrete tight junction proteins such as ZO-1 and occludin, thereby enhancing the tight junctions between epithelial cells, reducing intestinal permeability, and preventing harmful substances, bacteria, and toxins from entering the body, which decreases damage to the intestinal mucosa and inflammatory stimuli, consequently inhibiting the development of colorectal cancer (Kim, 2023; Jiang, 2024; Petruzziello et al., 2024). Research has confirmed that a study involving 30 pancreatic cancer patients divided them into a control group and a combination group, with the control group receiving enteral nutrition and the combination group receiving enteral nutrition plus probiotic supplementation. The results indicated that enteral nutrition combined with probiotic supplementation significantly protected the intestinal mucosal barrier in pancreatic cancer patients, with *Lactobacillus* rhamnosus promoting mucus secretion and forming a thicker and more protective mucus layer that obstructs the contact between pathogens and intestinal epithelial cells, thereby maintaining the integrity of the intestinal barrier (Wu et al., 2024).

#### 5.1.3 In terms of the mediation of metabolic products

The mechanisms by which gut microbiota exert anti-colorectal cancer effects through metabolites are complex and multifaceted, with short-chain fatty acids (SCFAs), secondary bile acids, and polyamines playing pivotal roles in regulating host physiological and pathological processes. SCFAs (e.g., butyrate, propionate, and acetate) are the primary end products of dietary fiber fermentation by gut microbiota. Butyrate induces histone hyperacetylation by inhibiting histone deacetylases (HDACs), thereby activating tumor suppressor genes (e.g., p21 and Bax) and promoting cell cycle arrest and apoptosis in colorectal cancer cells (Song et al., 2023). Additionally, butyrate inhibits the NF-κB signaling pathway by activating G protein-coupled receptors (GPR43 and GPR109A), reducing the release of pro-inflammatory cytokines (IL-6, TNF-α), and blocking chronic inflammation-driven carcinogenesis. Clinical studies have shown that the abundance of butyrate-producing bacteria (e.g., Roseburia) is significantly reduced in colorectal cancer patients, and supplementation with butyrate precursors (e.g., resistant starch) can restore SCFA levels and inhibit tumor growth (Zhang et al., 2024). Primary bile acids are converted into secondary bile acids (e.g., deoxycholic acid and lithocholic acid) by gut microbiota (e.g., Bacteroides), which exhibit cytotoxicity and genotoxicity at high concentrations, inducing DNA damage and oxidative stress. However, beneficial bacteria such as *Bifidobacterium* reduce secondary bile acid production by inhibiting 7α-dehydroxylase activity and promote their conjugation with taurine to form less toxic compounds for excretion (Ryu, 2020). Furthermore, secondary bile acids can regulate intestinal stem cell proliferation and differentiation by activating the farnesoid X receptor (FXR), thereby suppressing abnormal hyperplasia. Polyamines (e.g., putrescine and spermidine) are important microbial metabolites that promote intestinal epithelial repair at low concentrations but stimulate cell proliferation and tumor progression when excessively accumulated. Probiotics (e.g., *Lactobacillus* acidophilus) degrade excess polyamines by secreting polyamine oxidase, maintaining intestinal homeostasis. Studies have shown elevated fecal polyamine levels in colorectal cancer patients, and probiotic intervention significantly reduces their concentration, inhibiting tumor angiogenesis (Guven et al., 2020). Tryptophan is metabolized by microbiota into indole derivatives (e.g., indole-3-propionic acid), which activate the aryl hydrocarbon receptor (AhR), promote regulatory T cell differentiation, suppress Th17-mediated inflammatory responses, and enhance the efficacy of immune checkpoint inhibitors (e.g., anti-PD-1) (Zaragoza-García et al., 2020). Additionally, the activity of indoleamine 2,3-dioxygenase (IDO) is regulated by microbial metabolites, influencing tryptophan depletion and T cell function, thus providing new targets for combination therapy. In summary, microbial metabolites exert anti-colorectal cancer effects through multiple pathways, including epigenetic regulation, inflammation suppression, and immune modulation, making targeted metabolic pathways a promising direction for future precision.

### 5.2 Immunotherapy

**Figure 5:**
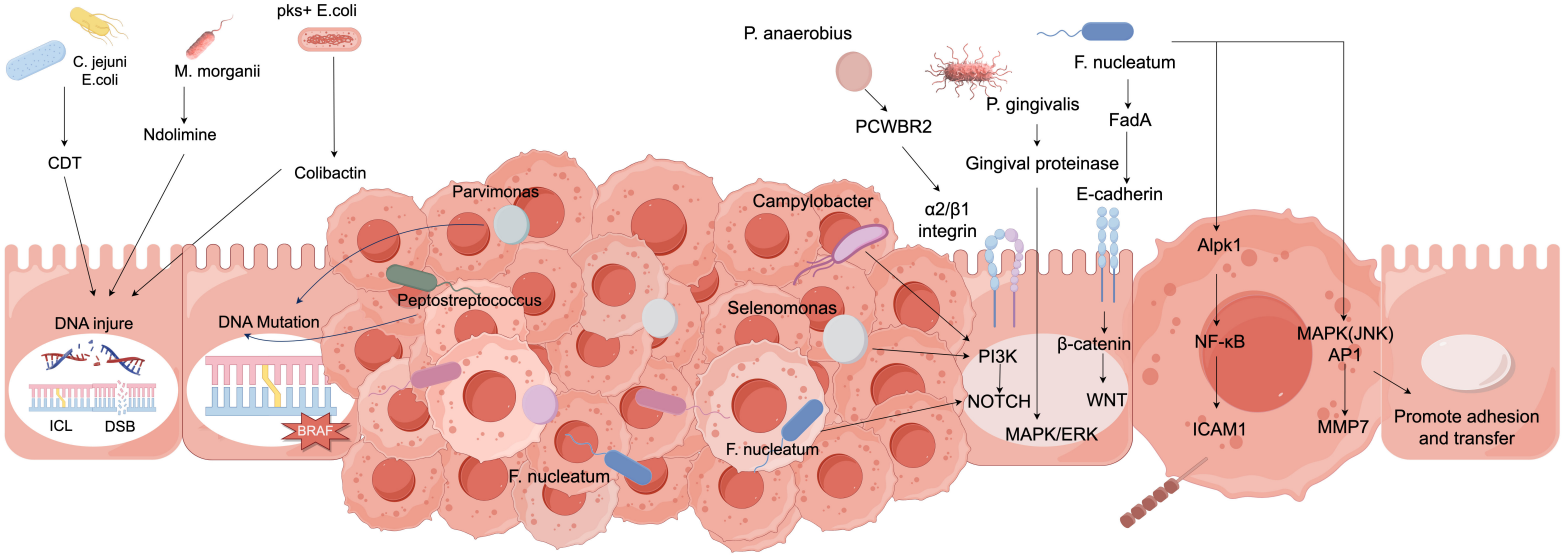
The relationship between gut microbiota and the occurrence, development, and treatment of colorectal cancer.

Immunotherapy has emerged as one of the important modalities for the treatment of colorectal cancer, with the gut microbiota playing a crucial role in this process. Immunotherapy is a treatment approach that utilizes the body’s own immune system to combat colorectal cancer. The core of this strategy lies in the activation and enhancement of the body’s immune cells, such as T cells and NK cells, enabling them to accurately recognize and attack colorectal cancer cells (Fan et al., 2021; Lindner et al., 2023; Marano et al., 2023; Park, 2024). See **Figure 5**.

#### 5.2.1 Regulation of immune cell function

**Figure 6:**
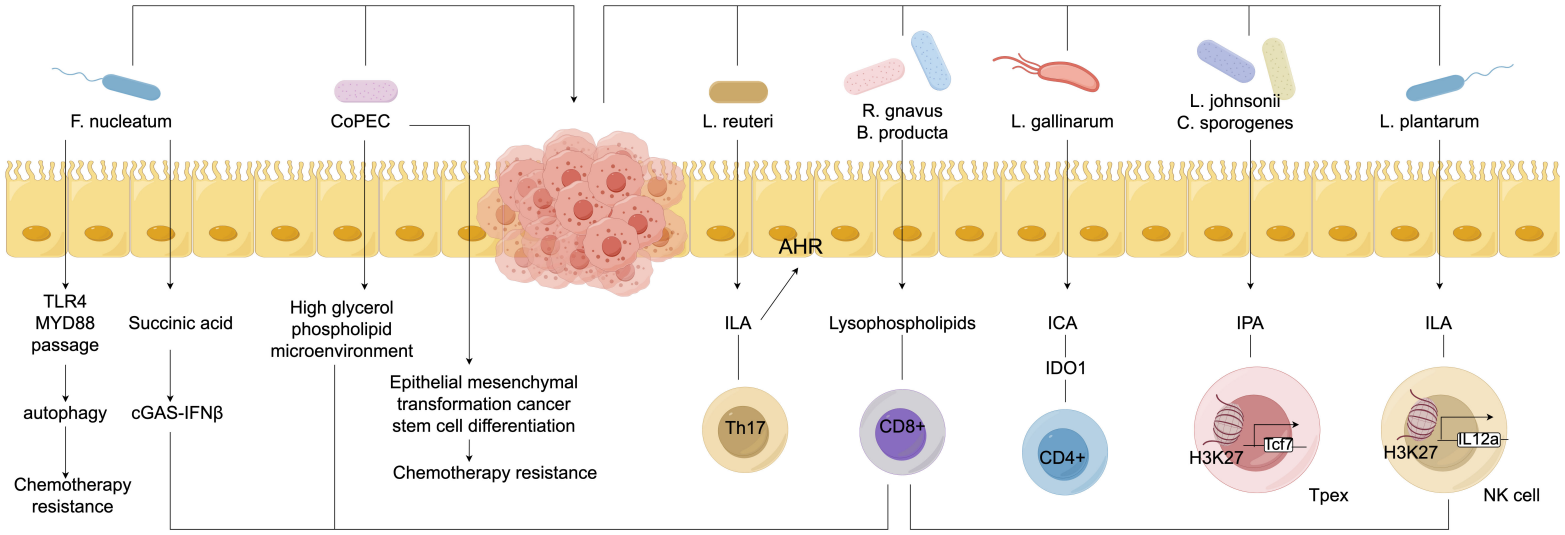
Diagram of molecules regulating immune cell function.

Gut microbiota can promote the maturation and activation of dendritic cells (DCs), increase the expression of surface co-stimulatory molecules, and enhance their antigen-presenting capacity, thereby more effectively activating cytotoxic T lymphocytes (CTLs) and helper T lymphocytes (Th), which improves their cytotoxic effects against colorectal cancer cells (Wu et al., 2022). Research has confirmed that fresh tumor tissues from clinical patients were collected to establish a colorectal cancer model in severely immunodeficient mice, which were divided into a blank group, a model group, and an immune modulation agent group. Analysis revealed that the immune function of the rats in the immune modulation agent group was significantly improved due to the presence of beneficial bacteria, such as *Bifidobacterium*, in the mice, which can modulate the signaling pathways within DCs, allowing for better recognition of tumor antigens and presentation to T cells, thereby stimulating an immune response (Carneiro et al., 2022). Additionally, the gut microbiota also influences the activity and cytotoxicity of natural killer (NK) cells (Li and Han, 2024). By secreting specific metabolic products or interacting with NK cell surface receptors, gut microbiota can regulate the functional state of NK cells, enhancing their ability to recognize and kill colorectal cancer cells, thereby strengthening the body’s anti-tumor immune defense in immunotherapy and improving therapeutic outcomes, providing new targets and research directions for immunotherapy in colorectal cancer (Dai et al., 2023; Sun et al., 2023; Huang B. et al., 2024). See **Figure 6**.

#### 5.2.2 Influence on the expression of immune checkpoint molecules

The immune checkpoint molecule programmed cell death receptor 1 (PD-1) and its ligand (PD-L1) are critical factors in tumor immune evasion. Research has confirmed that relevant scholars conducted foundational experiments and discovered PD-1 for the first time through interleukin-3 deprivation in mice (Li X. et al., 2022; Mafra et al., 2024). This protein is expressed in activated T/B lymphocytes. The gut microbiota can regulate signaling pathways within immune cells, thereby influencing the expression levels of these immune checkpoint molecules. Studies have demonstrated that transfecting the indoleamine 2,3-dioxygenase gene into mouse dendritic cells (DCs) and performing real-time quantitative polymerase chain reaction and immunocytochemical assays, designated into six groups: DC group, empty vector transfected DC group, transfected DC group, transfected DC + tryptophan group, DC + tryptophan metabolite group, and transfected DC + tryptophan metabolite group, revealed that transfected DCs can express functional indoleamine 2,3-dioxygenase gene, and that transfected DCs combined with tryptophan metabolites can synergistically inhibit CD4+ T cell proliferation (Feng et al., 2020; Kobayashi et al., 2021; Yu et al., 2022). It can thus be concluded that certain specific gut microbes, such as indole and tryptophan metabolites, can act on immune cells through their metabolic products to reduce the expression of PD-L1 on the surface of tumor cells, making it difficult for tumor cells to suppress the activity of immune cells, enhancing the cytotoxic capability of immune cells against tumor cells, and improving the efficacy of immune checkpoint inhibitors (Zhang et al., 2023; D’Alessio et al., 2024; Li et al., 2024). Furthermore, the gut microbiota may also influence the expression of other immune checkpoint molecules, such as cytotoxic T lymphocyte-associated antigen 4 (CTLA-4), thereby regulating the balance between the immune system and tumor cells from multiple dimensions, optimizing the effects of immunotherapy, and providing new research perspectives and potential therapeutic targets for immunotherapy in colorectal cancer (Pusceddu and Del Bas, 2020; Dikeocha et al., 2022; Ding et al., 2024).

#### 5.2.3 Immunoregulatory role of metabolites

Metabolites produced by the gut microbiota, such as short-chain fatty acids (SCFAs), play a significant regulatory role in immunotherapy. SCFAs, particularly butyrate, can inhibit histone deacetylase (HDAC) activity, thereby altering the gene expression profile of immune cells and enhancing their immune functions. Relevant studies have confirmed that butyrate promotes the differentiation and activation of T cells, enabling them to more effectively recognize and attack colorectal cancer cells (Guan and Liu, 2023; Yuan et al., 2024). Additionally, other research has demonstrated that butyrate can impact the barrier function of gastrointestinal epithelial cells under inflammatory conditions, leading to a loss of function (Okumura et al., 2021; Grosicki et al., 2023; Kang et al., 2023). Furthermore, SCFAs can modulate the inflammatory response, reducing the release of pro-inflammatory factors and creating a microenvironment conducive to immunotherapy, thereby enhancing the infiltration and cytotoxicity of immune cells against tumors. This synergistic effect with immunotherapy improves the treatment efficacy for colorectal cancer, providing new insights for optimizing immunotherapeutic strategies and revealing the critical role of gut microbiota metabolites in immune regulation and colorectal cancer treatment.

#### 5.2.4 Regulation of the tumor microenvironment

**Figure 7:**
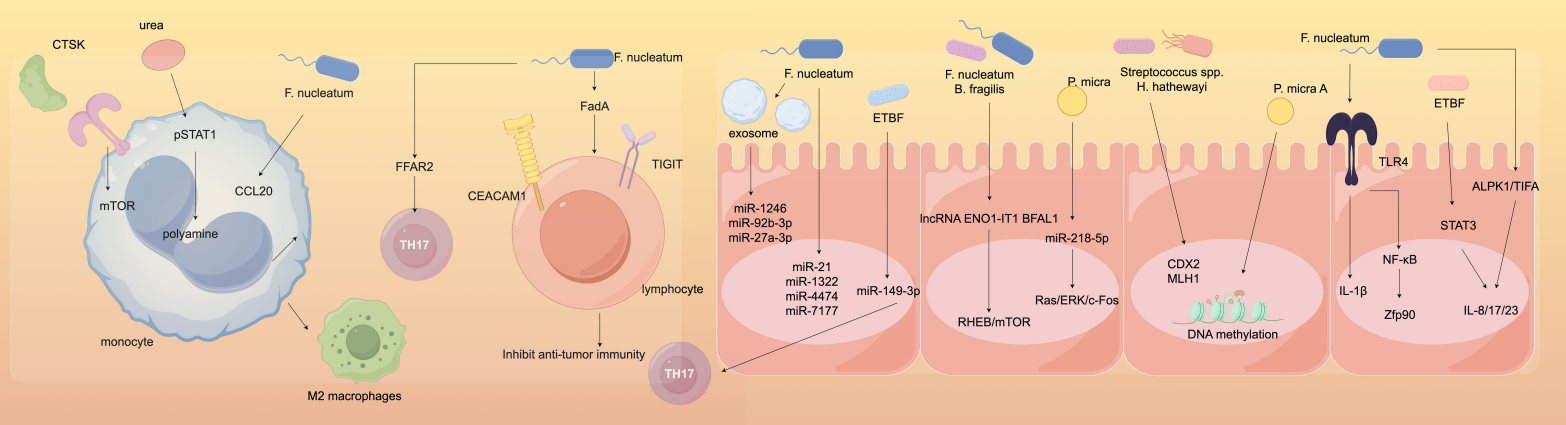
Molecular diagram of tumor microenvironment regulation.

On one hand, the microbiota can modulate the cytokine network within the tumor microenvironment. Beneficial bacteria can stimulate immune cells to secrete cytokines such as interferon-γ and tumor necrosis factor-α, enhancing the anti-tumor activity of immune cells while simultaneously inhibiting the production of immunosuppressive factors such as interleukin-10 and transforming growth factor-β, thereby reducing immunosuppressive signals within the tumor microenvironment. On the other hand, gut microbiota influence the expression and secretion of chemokines (Tanes et al., 2021; Liang et al., 2022; Guo et al., 2023). Relevant studies have confirmed that the upregulation of chemokines such as CXCL9 and CXCL10, which are microbial metabolites, can induce corresponding tumor tissues in colorectal cancer, attracting more cytotoxic T lymphocytes and natural killer cells to infiltrate the tumor site, thereby enhancing the cytotoxic effects of immune cells against tumor cells, remodeling the tumor microenvironment from an immunosuppressive state to an immune-activated state, and improving the efficacy of immunotherapy for colorectal cancer, thus opening new avenues for comprehensive treatment of colorectal cancer (Marzhoseyni et al., 2024). See **Figure 7**.

### 5.3 Fecal microbiota transplantation

Fecal microbiota transplantation (FMT), as an emerging therapeutic approach, exhibits a unique mechanism of action in the association between gut microbiota and the efficacy of colorectal cancer treatment, offering new insights and hope for the management of colorectal cancer (Chi et al., 2021; Liu Y. et al., 2022; Saygili et al., 2024). The impact of FMT on the efficacy of colorectal cancer treatment is primarily achieved through the restructuring of the gut microbiota composition. The feces of healthy donors contain a rich and diverse microbial community, which, upon transplantation into patients, can rectify the dysbiotic state of the gut microbiota in colorectal cancer patients (Halverson and Alagiakrishnan, 2020; Guo et al., 2022; Nabizadeh et al., 2023; Hammer et al., 2024). Studies have confirmed that beneficial bacteria with anti-cancer properties, such as butyrate-producing bacteria and bifidobacteria, may recolonize and proliferate significantly within the recipient’s gut, inhibiting the growth of harmful bacteria and optimizing the gut microbial ecosystem, thereby creating favorable conditions for subsequent anti-cancer therapies (Ding and Xiao, 2020; Młynarska et al., 2022; Lloyd, 2024).

#### 5.3.1 Immunomodulation aspect

Fecal microbiota transplantation (FMT) can significantly influence the immune response of the host. Clinical studies have demonstrated that the transplanted gut microbiota can modulate the function of gut-associated lymphoid tissue, leading to alterations in the activity and function of immune cells such as T lymphocytes and natural killer cells (Polak et al., 2021; Calabrò et al., 2023; Chasov et al., 2024). Beneficial bacteria can enhance the antigen-presenting capacity of dendritic cells by stimulating their maturation and activation, thereby activating cytotoxic T lymphocytes to exert cytotoxic effects against colorectal cancer cells (Xu et al., 2024; Loo et al., 2020; Sasso et al., 2023; Su et al., 2024). Concurrently, FMT may also regulate the cytokine secretion profile, inhibiting the excessive production of inflammatory factors such as tumor necrosis factor-α and interleukin-6, alleviating intestinal inflammatory responses, enhancing the host’s anti-tumor immune response, and improving the efficacy of immunotherapeutic agents (Zhao et al., 2022; Bernabeu et al., 2024; Wang et al., 2024).

#### 5.3.2 Metabolic pathways aspect

Fecal microbiota transplantation (FMT) involves the transfer of functional microbial communities from healthy donor feces into the intestinal tract of patients to reconstruct gut microbiota homeostasis. Colorectal cancer patients typically exhibit dysbiosis characterized by increased pathogenic bacteria and diminished beneficial species. FMT enhances the abundance and diversity of beneficial intestinal flora such as *Bifidobacterium* and *Lactobacillus*, which participate in multiple metabolic pathways. Post-FMT intervention, gut microbiota metabolizes dietary fibers to produce short-chain fatty acids (SCFAs) including acetate, propionate, and butyrate. Animal studies demonstrate that FMT-treated mice exhibit 2.5-fold elevation in colonic butyrate concentrations and 40% reduction in tumor volume (Kim and Lee, 2021; Li Z. et al., 2022; Kong et al., 2023). As the primary energy substrate for colonocytes, butyrate promotes normal cellular proliferation and differentiation while exhibiting inhibitory effects on colorectal carcinogenesis (Gou et al., 2023; Huang et al., 2023; Ionescu et al., 2023). Concurrently, SCFAs modulate intestinal pH levels to suppress pathogenic bacterial growth and maintain mucosal microenvironment stability. Gut microbiota participates in bile acid biotransformation, with FMT regulating metabolic pathways to enhance conversion of primary bile acids into secondary forms. Certain secondary bile acids like lithocholic acid demonstrate carcinogenic potential, whereas FMT-mediated metabolic modulation alters bile acid composition ratios, reducing oncogenic derivatives and mitigating colorectal cancer risk. Clinical investigations reveal post-FMT elevation in fecal taurocholic acid proportions negatively correlating with tumor marker (CEA) levels (Wei et al., 2024). FMT modulates tryptophan metabolism pathways, promoting generation of indole derivatives that activate aryl hydrocarbon receptors. These metabolites regulate immune responses, enhance epithelial barrier integrity, and remodel tumor immune microenvironments, thereby inhibiting neoplastic progression and metastasis (Gamez-Belmonte et al., 2023; Png et al., 2024). Collectively, FMT orchestrates multidimensional metabolic reprogramming to reconstruct intestinal microenvironments, offering novel therapeutic strategies for colorectal cancer management through metabolic pathway regulation.

#### 5.3.3 Tumor microenvironment aspect

Following colonization in the gut, transplanted healthy fecal microbiota alter the composition and metabolites of the intestinal microbiome, indirectly influencing the tumor microenvironment (Chen H. et al., 2023; Chen S. et al., 2023; Lu et al., 2024). Metabolites produced by beneficial bacteria modulate cytokine levels in the tumor microenvironment, reducing the secretion of pro-tumorigenic cytokines such as IL-6 and TNF-α while increasing the release of anti-tumor cytokines like IFN-γ, thereby inhibiting tumor cell proliferation and metastasis (Cho et al., 2020; Dong et al., 2022; Zheng et al., 2024). Additionally, fecal microbiota transplantation (FMT) impacts immune cell infiltration and function within the tumor microenvironment. Studies have shown (Katoh and Katoh, 2022) that FMT promotes the infiltration of more CD8+ T lymphocytes and natural killer cells into tumor tissues, enhancing their cytotoxic activity against tumor cells, while suppressing the function of immunosuppressive cells such as regulatory T cells. This reshapes the immune balance in the tumor microenvironment, shifting it from a pro-tumor to an anti-tumor state, improving the therapeutic efficacy of colorectal cancer, and opening new avenues for its comprehensive treatment.

### 5.4 The beneficial role of microbiota in preventing adverse events associated with radiotherapy and chemotherapy for colorectal cancer

Radiotherapy and chemotherapy are the cornerstone treatments for Colorectal Cancer (CRC), yet their adverse effects, including gastrointestinal toxicity, immunosuppression, and secondary infections, severely limit therapeutic efficacy and decrease patients’ quality of life. Recent studies have shown that the gut microbiota alleviates these treatment-related adverse events through multidimensional mechanisms, with specific modes of action as follows:

#### 5.4.1 Maintaining intestinal barrier function: resisting pathogen invasion and inflammation spread

Chemotherapeutic drugs (such as 5-fluorouracil and irinotecan) and radiotherapy can induce intestinal epithelial cell apoptosis and disrupt tight junction proteins (e.g., ZO-1, occludin), leading to “leaky gut,” which facilitates the translocation of intestinal endotoxins (e.g., lipopolysaccharide) into the bloodstream, triggering systemic inflammatory responses and even sepsis. The gut microbiota repairs barrier function through the following pathways: Direct protective effects of probiotics: Bifidobacteria and lactobacilli stimulate intestinal epithelial cell proliferation and migration by activating the epidermal growth factor receptor (EGFR) signaling pathway, accelerating mucosal repair. For example, the extracellular polysaccharides secreted by *Lactobacillus* reuteri upregulate occludin expression and reduce irinotecan-induced intestinal permeability increases (Dariya et al., 2020). Barrier-enhancing effects of short-chain fatty acids (SCFAs): Butyrate promotes goblet cell secretion of mucin MUC2 by inhibiting histone deacetylase (HDAC), forming a dense mucus layer that physically isolates pathogens from epithelial contact. Animal experiments show that supplementing with butyrate precursors (resistant starch) increases the mucus layer thickness by 50% in irradiated mice, significantly reducing the risk of bacterial translocation (Liu et al., 2021b). Inhibition of pro-inflammatory cytokine release: Probiotics (e.g., *Lactobacillus* acidophilus) downregulate the nuclear factor-κB (NF-κB) pathway, reducing the production of tumor necrosis factor-α (TNF-α) and interleukin-6 (IL-6), blocking secondary damage to the intestinal barrier by inflammatory cascades. Clinical evidence: A randomized controlled trial involving 200 CRC chemotherapy patients showed that combined use of bifidobacteria and fructooligosaccharides (prebiotics) reduced the incidence of grade 3 or higher diarrhea from 42% to 18% (P<0.01), with a 60% decrease in serum endotoxin levels (Wong et al., 2020).

#### 5.4.2 Regulating immune homeostasis: balancing inflammation and immune reconstruction

Radiotherapy and chemotherapy often lead to bone marrow suppression and lymphocyte depletion, increasing the risk of opportunistic infections. The gut microbiota restores immune homeostasis through the following mechanisms: Enhancing innate immune responses: *Bifidobacterium* enhances the phagocytic function of neutrophils and macrophages by activating Toll-like receptor 2 (TLR2) and promoting the secretion of interferon-γ (IFN-γ) by dendritic cells (DCs). For example, in a mouse model of radiotherapy, supplementation with *Bifidobacterium* longum restored neutrophil counts to 80% of normal levels, significantly reducing sepsis mortality (Reis et al., 2019). Regulating adaptive immunity: Butyrate induces the differentiation of regulatory T cells (Tregs) by activating the GPR43 receptor, inhibiting excessive inflammatory responses. Meanwhile, propionate reduces IL-17-mediated intestinal damage by inhibiting Th17 cell differentiation. Clinical studies have shown a positive correlation between butyrate concentration in the feces of CRC patients and the proportion of Tregs in peripheral blood (r=0.71), suggesting its immune-modulating potential (Erfanian et al., 2023). Improving the efficacy of immune checkpoint inhibitors (ICIs): The gut microbiota, such as butyrate-producing bacteria, enhances the antitumor effect of ICIs by upregulating the sensitivity of the PD-1/PD-L1 signaling pathway. A retrospective analysis found that among CRC patients receiving anti-PD-1 treatment, those with a higher abundance of Faecalibacterium in the gut had an objective response rate (ORR) of 45%, significantly higher than that of the low-abundance group (15%) (Biondi et al., 2021).

#### 5.4.3 Metabolic detoxification and antioxidation: reducing therapeutic toxicity

Chemotherapeutic agents and radiotherapy generate reactive oxygen species (ROS) and toxic metabolites, which can exacerbate tissue damage. The gut microbiota exerts a detoxifying effect through the following pathways: Drug metabolism and transformation: Certain Clostridium species, such as Clostridium butyricum, express β-glucuronidase, which reconverts the toxic metabolite SN-38G of irinotecan into its inactive form, thereby reducing intestinal epithelial cell damage. Animal experiments have shown that supplementation with C. butyricum can reduce intestinal SN-38 concentration by 70% and decrease the incidence of mucositis by 50% (Ghanavati et al., 2020; An et al., 2023; Cho et al., 2024). Antioxidant defense: Probiotics, such as *Lactobacillus* plantarum, neutralize free radicals generated by radiotherapy by secreting glutathione (GSH) and superoxide dismutase (SOD). *In vitro* studies have demonstrated that the supernatant of *Lactobacillus* plantarum can reduce the apoptosis rate of radiation-induced intestinal epithelial cells from 35% to 12% (Garavaglia et al., 2022). Bile acid metabolism regulation: Secondary bile acids, such as deoxycholic acid, can induce intestinal epithelial apoptosis by activating the farnesoid X receptor (FXR). However, *Bifidobacterium* reduces the production of secondary bile acids by inhibiting 7α-dehydroxylase activity. Clinical trials have confirmed that probiotic intervention can lower deoxycholic acid levels in the feces of CRC patients by 40%, which is significantly correlated with improved mucosal injury scores (Liu X. et al., 2022).

#### 5.4.4 Inhibiting opportunistic pathogens: reestablishing ecological balance of gut microbiota

Antibiotic overuse and immunosuppression readily lead to overproliferation of pathogens such as Clostridioides difficile, while microbiota intervention can restore ecological balance through the following mechanisms: Competitive exclusion: Fecal microbiota transplantation (FMT) introduces butyrate-producing bacteria (e.g., members of the genus Roseburia) to competitively inhibit the colonization of C. difficile. A multicenter study showed that FMT achieved a cure rate of 92% for recurrent C. difficile infections, significantly outperforming vancomycin (67%) (Benito et al., 2021; Barreira et al., 2023). Antimicrobial substance secretion: Lactic acid bacteria directly kill drug-resistant bacteria by producing bacteriocins (e.g., reuterin). *In vitro* experiments demonstrated that reuterin secreted by *Lactobacillus* reuteri could eliminate 90% of methicillin-resistant Staphylococcus aureus (MRSA) within 6 hours (Chen et al., 2020; Sharma and Shukla, 2020; Rohani et al., 2024). Metabolite inhibition of pathogens: Butyrate inhibits the growth of pathogenic bacteria (e.g., Salmonella) by lowering intestinal pH. Additionally, the antimicrobial properties of secondary bile acids can selectively eliminate some Gram-negative bacteria, maintaining microbial stability.

## 6 Conclusion and prospects

### 6.1 Conclusion

The intestinal microbiota exhibits intricate and pivotal mechanisms in combating colorectal carcinogenesis. On one hand, beneficial microorganisms enhance immune surveillance through immunomodulation, activating effector cells to improve neoplastic cell recognition and cytotoxicity, while simultaneously suppressing pro-inflammatory cascades and reducing pro-carcinogenic factor production. On the other hand, microbial metabolites such as short-chain fatty acids (SCFAs) exert anticancer effects by modulating cellular proliferation, apoptosis, and epigenetic modifications. Multi-omics technologies provide robust methodologies for mechanistic exploration: metagenomics enables comprehensive profiling of microbial genetic architecture to identify potential anticarcinogenic functional genes; metabolomics captures dynamic metabolite flux to pinpoint critical therapeutic biomolecules; while transcriptomics and proteomics delineate molecular cross-talk between microbial communities and host cells at gene expression and protein interaction levels. Current research limitations persist, particularly in elucidating the tripartite interaction network encompassing gut microbiota-host-environment crosstalk, with mechanistic pathways of specific microbial taxa and their metabolic derivatives remaining partially characterized.

### 6.2 Future prospects

With the advancement of multi-omics technologies, single-cell sequencing, and bioinformatics, there is a promising prospect for further elucidating the intricate molecular interaction networks between the gut microbiome and colorectal cancer. This progress may facilitate the realization of personalized diagnostics and precision therapies based on the gut microbiome, thereby offering new hope for improving the prognosis and quality of life of colorectal cancer patients and propelling the prevention and treatment of colorectal cancer to new heights.
